# Carob Seed Peels Effect on Cognitive Impairment and Oxidative Stress Status in Methionine-Induced Mice Models of Schizophrenia

**DOI:** 10.3390/brainsci12121660

**Published:** 2022-12-03

**Authors:** Imane Lakkab, Abdelmoughite Ouakil, Hanane El Hajaji, Nadya Lachkar, Radu Lefter, Alin Ciobica, Brahim El Bali, Romeo Dobrin, Luminita Diana Hritcu, Mohammed Lachkar

**Affiliations:** 1 Engineering Laboratory of Organometallic, Molecular Materials, and Environment, Faculty of Sciences, Sidi Mohammed Ben Abdellah University, Fez 30000, Morocco; 2 Center of Biomedical Research, Romanian Academy, Iasi, B dul Carol I, No 8, 010071 Bucharest, Romania; 3 Department of Research, Faculty of Biology, Alexandru Ioan Cuza University of Iasi, Bd. Carol I, 20A, 700505 Iasi, Romania; 4 Independent Scientist, Oujda 60000, Morocco; 5 Faculty of Medicine, Grigore T. Popa University of Medicine and Pharmacy, Strada Universitatii 16, 700115 Iasi, Romania; 6 Internal Medicine Clinic, Ion Ionescu from Brad University of Life Sciences, 3 Sadoveanu Alley, 700490 Iasi, Romania

**Keywords:** *Ceratonia siliqua* L., antioxidant activity, schizophrenia, depression and anxiety, antioxidant enzymes

## Abstract

**Background:***Ceratonia siliqua L.* (Carob tree) is a Mediterranean evergreen, well known for its medicinal properties. The different parts of Carob were proven to exert antidiabetic, antibacterial, antifungal, and antiproliferative effects. Hence, the present paper aims to validate the positive correlation between the high antioxidant activity of carob seed peels and the improvement of negative symptoms of schizophrenia. **Materials & Methods:** The antioxidant activity was carried out using the β-carotene test. Methionine and carob seed peels (CSP) extracts (50 and 100 mg/kg) were orally administrated to mice for a week. After administration, behavioral tests were assessed using the Y-maze, elevated plus maze, and forced swimming tests, as well as the novel object recognition task. Furthermore, the oxidative stress status was evaluated by analyzing the levels of the antioxidant enzymes: Superoxide dismutase (SOD), glutathione peroxidase (GPx), and malondialdehyde levels (MDA). **Results:** Both extracts exhibited remarkable antioxidant activity and showed antibacterial effect against Gram-positive bacteria tested (*Bacillus subtilis* and *Staphylococcus aureus*) and against *Pseudomonas aeruginosa* (Gram-negative). Therefore, *Escherichia coli* was very resistant. The behavioral tests proved the efficacy of CSP in enhancing the cognitive impairment of animal models of schizophrenia. Hence, the stated correlation between oxidative stress and schizophrenia was confirmed by the increased SOD and GPx activities and the decreased MDA level. **Conclusions:** The present study gave further confirmation of the potential correlation between oxidative stress and the development of psychiatric disorders and highlighted the use of natural antioxidants, especially *Ceratonia siliqua* L. in the improvement of cognitive impairment in the dementia of schizophrenia.

## 1. Introduction

Schizophrenia is a chronic neuropsychiatric disorder distinguished by trouble with thinking, speaking, and memory, split personality, hallucination, disordered behavior, and recently, trouble reading was added to the list of schizophrenia symptoms [1]. More than that, the risk for suicide, cardiovascular diseases, and also breast cancer were reported to be important in schizophrenia patients [2]. In the most recent study reporting the statistical rate of schizophrenia in the world, around 21 million people are diagnosed living with schizophrenia. While the majority of the highest rates are located in lower- and moderate-income countries [3]. Taking into consideration the financial charges of the medical treatment of schizophrenia, the exploration of new pathways of treatment is mandatory. In light of this objective, the hypothesis of the implication of oxidative stress in schizophrenia disorder appears to propose a promising approach to underline the mechanisms of the development of this dementia and provide new therapeutic strategies.

Oxidative stress is defined as an imbalance between reactive oxygen species (ROS) and antioxidants. Excess production of ROS leads to adverse effects on essential cellular components such as DNA, proteins, and lipids, leading to a range of serious pathologies and diseases [4]. Consequently, to prevent oxidation and regulate or inhibit the excessive production of ROS, antioxidants, including endogenous and exogenous ones, intervene according to their mechanisms of action and compensate for this imbalance [5,6].

Superoxide dismutase (SOD), glutathione peroxidase (GPx), and Malondialdehyde-MDA are the principal endogenous enzymatic markers of oxidative stress widely used to demonstrate the effect of oxidative stress damage on the development of various disorders and diseases [7]. The growing body of literature has evaluated the level of these oxidative stress markers related to Schizophrenia and confirmed their significant dysregulation. Several studies reported an increased level of SOD [8,9], while others have demonstrated a significant decrease level of SOD and of GPx-specific activities [10,11,12,13,14]. Meanwhile, the majority of the studies demonstrated a significant increase in the lipid peroxidation marker (Malondialdehyde-MDA) levels [9,12,13]. Inboard, all these studies reach the conclusion of the involvement of OS damages on the development of schizophrenia.

Modern medicine uses treatments based on synthetic molecules such as neuroleptics, anxiolytics, and antidepressants to treat neuropsychiatric diseases such as schizophrenia. Certainly, these treatments can exert some positive effects to decrease the degree of the severity of these disorders, but at the same time, they have important side effects which can be dangerous in certain cases [15,16]. Based on this fact, the present study comes to remedy these drawbacks and to frame the use of aromatic and medicinal plants for the treatment of these diseases.

The carob tree (*Ceratonia siliqua* L.) is an endemic evergreen species widespread in the Mediterranean area and belongs to the pea family, *Fabaceae* [17]. Portugal, Italy, and Morocco are the top main world producers of carob (FAOSTAT, 2017). The most commonly used parts of the carob tree are the pods for their high carbohydrate and polyphenol content and the seeds for the carob gum (LBG). In addition, the carob tree has been investigated by various phytochemical pieces of research on almost all the parts of the tree (roots, bark, leaves, and fruit) and has been identified as rich in organic substances with high added value, especially in the pharmacological field. In fact, the leaves were reported to exert antidiarrheal, antidiabetic, and antiproliferative effects against the hepatocellular carcinoma cell line [18,19]. The pods were found to be strong as antidiabetic agents and proved their efficiency against gastric lesions and ulcers [20,21]. Other researchers suggested the germ of carob as a cytotoxic agent [11,14]. Alzoubi et al. found that the methanol extract from carob pods prevented stress-induced memory impairment [22]. Moreover, a recent study by Lakkab et al. [23] confirmed the potential anxiolytic and antidepressant activities of carob seeds peels (CSP) extracts. Thus, this study highlighted the capacity of CSP to act as a natural agent in the treatment of mood disorders.

In recently published research, studies of different parts of the carob tree revealed their richness in bioactive compounds with high antioxidant activity. Nevertheless, the present paper aims to validate the effectiveness of CSP as a natural antioxidant compound and sheds new light on the potential protective effect of CSP against negative symptoms of mice models of schizophrenia induced by the administration of methionine.

## 2. Materials and Methods

### 2.1. Sample Preparation

The carob seed samples used in this study came from the northern and southern regions of Morocco. Voucher specimens were previously identified by Professor A. Ennabili and have been deposited in the Herbarium of the National Institute of Medicinal and Aromatic Plants, Sidi Mohamed Ben Abdellah University, Fez, Morocco [24]. The seeds were pretreated using the enzymatic hydrolysis method, and the different parts were manually separated [25]. Then, the peels were dried and milled, and the resulting powder was stored at 4 °C for further experiments.

### 2.2. Plant Material and Preparation of Extracts

Ethyl acetate and acetone extracts were obtained by sequential extraction with solvents, according to the ascending order of their polarity. Plant material was mixed with 100 mL of ethyl acetate solvent and stored at room temperature in a flask. After 24 h, the solution was filtered through a Whatman filter paper (#1), and the residue was re-extracted with an equal volume of the same solvent. The process was repeated after 48 h using 70:30 acetone (acetone: water). The recovered filtrates were evaporated under a vacuum at the boiling temperature of each solvent used by a rotary evaporator and stored at 4 °C.

### 2.3. Total Phenolic Content

The method described by Singleton et al. was carried out to measure the total phenolic content with slight changes [26]. 200 µL of each extract with a determined concentration of (0.5 mg/mL) was mixed with 1 mL of Folin-Ciocalteu reagent and 800 µL of sodium carbonate (7.5%). After 30 min, the concentration of total phenolic content was estimated at Gallic acid equivalent (GAE) and expressed as mg GAE/g of extract. The absorbance was measured at 765 nm using a UV-VIS spectrophotometer.

### 2.4. Total Flavonoid Content

The total flavonoid content of each extract of CSP was measured using the method of aluminum trichloride (AlCl3) [27]. Briefly, a concentration of (0.5 mg/mL) was added to an equal volume of AlCl3 (2%). The mixture was then stirred and incubated for 30 min. The absorbance was then read at 415 nm, and the results were expressed using a calibration curve of Quercetin equivalent (µg Qe E/g extract).

### 2.5. Antibacterial Activity

*Bacillus subtilis* ILP1428B, *Staphylococcus aureus* CIP543154, *Pseudomonas aeruginosa* ATCC27653, and *Escherichia coli* CIP5412 were used to test the antibacterial activity of both extracts of *Ceratonia siliqua* L. seed peels. The strains were obtained from the Laboratory of Microbial Biotechnology, Faculty of Sciences and Technologies, University Sidi Mohammed Ben Abdellah, Morocco. The antibacterial activity evaluation was performed according to a previously described methodology of Bouhdid et al. [28], with some adjustments. All bacterial strains were cultured at 37 °C on Luria-Bertani broth (LB) for 24 h. The minimum inhibitory concentration (MIC) was determined using the microdilution assay. Samples of CSP extracts were adjusted to a known concentration of (100 mg/mL) diluted in 2% of DMSO, and distributed in the microdilution plates, 50 µL of bacteria inoculum (10^6^ CFU/mL) were added. Microplates were incubated at 37 °C for 24 h. Then, resazurin (10 μL) was added for color development. Bacterial growth is highlighted by the change of color from the blue dye resazurin to pink resorufin. The value of the first concentration that had the absence of blue color was recorded as MIC. The Minimum Bactericidal Concentration (MBC) is measured as the lowest concentration of the extracts resulting in negative subcultures after incubation at 37 °C for 24 h on LB plates. DMSO 2% was used as the negative control.

### 2.6. Antioxidant Activity by β-Carotene Bleaching Test

The antioxidant activity of CSP extracts was estimated using the procedure of Taga et al., and Barros et al. [29,30], with some changes. Briefly, 1 mg of β-carotene was mixed with 10 mL of chloroform, then 25 µL of linoleic acid and 200 mg Tween 40 were mixed in. The chloroform was next evaporated at 40 °C on a rotary evaporator. Then 100 mL of deionized water was slowly added to the mixture with vigorous stirring until the formation of an emulsion. There was 5 mL of the emulsion was mixed with 200 µL of extract prepared at a concentration of 100 µg/mL and incubated at 50 °C. Butylated hydroxytoluene (BHT) was used as an antioxidant of reference. The absorbance was measured at 470 nm using a spectrophotometer UV/Visible. After 48 h, the obtained results were compared with those of BHT. Tests were carried out in triplicate. The lipid peroxidation (LPO) inhibition was calculated following the following equation: % LPO inhibition = (β-carotene content after 48 h of assay/initial β-carotene content) × 100.

### 2.7. Behavioral Tests

#### 2.7.1. Animals

30 Male mice (6 mice per group) weighing 20–27 g at the start of the experiment. They were housed in a temperature and light-controlled room (22 °C, a 12-h cycle starting at 08:00 h) and were fed and allowed to drink water ad libitum. Mice were treated in accordance with the guidelines of animal bioethics from Directive 2010/63/EU of the European Parliament and of the Council of 22 September 2010. This study was approved by the local USAMV Ethics Committee (no. USAMV Iasi 385/04.04.2019).

#### 2.7.2. Drug Administrations

The animals were distributed into 6 groups. Group I: was administered saline solution and submitted to tests as the control group. Group II: administrated with methionine (37.5 mg/mL) and served as a model group for schizophrenia. The other 4 groups (EtOAc 50, EtOAc 100, Acet 50, and Acet 100) were daily subjected to a subcutaneous injection of 50 µL of methionine (37.5 mg/mL) (Sigma, USA) and orally administrated with extracts during 7 days. Accordingly, the groups of EtOAc 50 and Acet 50 (received methionine + a dose of 50 mg/kg of ethyl acetate and acetone extracts) and groups of EtOAc 100 and Acet 100 (received methionine + a dose of 100 mg/kg of the two extracts). The oral administration of extracts started from the 8th day of methionine injection. Oral administration of the extracts began on day 8, at the end of the methionine injection.

The method used for the period of treatment and time of pretest administration was established based on our pilot studies and prior published reports on animal models of schizophrenia [31,32].

#### 2.7.3. Y-Maze Test

The Y-maze task is made of three arms (35 cm long, 25 cm high, and 10 cm wide) with an equilateral triangular central area [33,34]. The test is designed to study short-term memory by assessing spontaneous alternation behavior. The mouse is placed in a central equilateral zone and allowed to move freely through the arms of the maze for 8 min. Spontaneous alternation behavior was defined as the complete entry of the mouse’s hind paws into each arm of the maze on consecutive choices. The number of maximum spontaneous alternation percentage behaviors was then calculated following the formula Equation (1):

(1) $$\mathrm{Spontaneous}\;\mathrm{alternation}\%=\frac{Spontaneous\;alternation}{total\;number\;of\;arm\;entries-2}\times 100$$

#### 2.7.4. Elevated Plus Maze

The Elevated Maze Test (EPM) is mainly used to screen anxious behavior. The EPM comprises four arms, 49 cm long and 10 cm wide, elevated 50 cm from the floor. Two arms are framed by 30 cm high barriers, and the other two arms are left open. A mouse was placed at the intersection of the four arms of the maze, facing an open arm, and the time passed on the open arms was registered during a 5-min test [34,35]. After each trial, the maze was cleaned with 70% to eliminate odors. To measure the index of anxiety-like behavior of all the distance traversed, the count of entrances into every arm, the time passed in every arm, and the percentage of entrances into the open arms are counted. [36].

#### 2.7.5. Forced Swimming Test

The procedure used to evaluate the antidepressant effect of CSP extracts is as reported by Detke et al. and Petit-Demouliere et al. [37,38]. On the 1st day of the experiments (pre-test session), each mouse was singly placed in cylindrical containers (diameter 30 cm, height 59 cm) filled with 25 cm of water at 26 ± 1 °C. Mice were permitted to swim for 6 min before being removed, dried, and replaced in their cages. The process was repeated 24 h later in a 6-min swimming session (test session). In the testing Session, the main behavior responses were recorded after 2 min of swimming (only the last 4 min was counted): the immobility time (the time that the mouse keeps floating with its head out of the water) and the time spent swimming.

#### 2.7.6. Novel Object Recognition

The novel object recognition test used in this study consists of measuring the memory recognition of a familiar wooden object and a new metallic one with the same sizes of 9 cm in length, 4 cm in width, and 4 cm in height. This task allows us to test the alteration of memory and learning, as well as to assess attention, anxiety, and novelty preference in the animals. [39]. After two days of habituation with two identical objects, on the third day, the mice were placed in an apparatus facing both objects: the familiar and the new one. The novel object preference was evaluated by recording the time spent checking the novel and familiar objects.

### 2.8. Tissue Collection

At the end of the behavioral tests, all mice were anesthetized and quickly dissected, and the complete brain was detached. The temporal lobes were collected, and each sample was weighed and homogenized with a Potter Homogenizer coupled with Cole-Parmer Servodyne Mixer (1 g tissue/10 mL of bidistilled water). Samples were centrifuged for 15 min at 3000 rpm, then the supernatant was isolated and pipetted into tubes.

### 2.9. Estimation of Antioxidant Enzymes

#### 2.9.1. Determination of Superoxide Dismutase

Superoxide dismutase (SOD) activity was estimated by the percent inhibition rate of the enzyme’s reaction with the substrate WST-1 (a water-soluble tetrazolium dye) and xanthine oxidase using a SOD assay kit (Fluka, product number: 19160) according to the manufacturer’s instructions. After 20 min, at 37 °C, the absorbance of the colored product of the reaction of WST-1 with superoxide was measured at 450 nm. Percent inhibition was expressed per mg of protein and presented in units of SOD activity [40].

#### 2.9.2. Determination of Glutathione Peroxidase

The glutathione peroxidase (GPx) cellular activity kit (from Sigma chemicals) was used to evaluate the GPx. This kit is based on an indirect method, consisting of the oxidation of glutathione (GSH) to oxidized glutathione (GSSG) catalyzed by GPx, which is then coupled with recycling GSSG back to GSH utilizing glutathione reductase (GR) and NADPH. The absorbance of the solutions was recorded at 340 nm. Thus, a decrease in NADPH during the oxidation to NADP is indicative of GPx activity [40].

#### 2.9.3. Determination of Malondialdehyde

Malondialdehyde (MDA) levels were measured by thiobarbituric acid reactive substances (TBARs) assay. Briefly, 200 μL of supernatant was added to 1 mL of 50% trichloroacetic acid, 0.9 mL of Tris-HCl (pH 7.4), and 1 mL of 0.73% thiobarbituric acid. For 20 min, the samples were homogenized and stirred gently with a vortex. Afterward, samples were centrifuged at 3000 rpm for 10 min. The signal was read against an MDA standard curve at 532 nm, and the results were expressed as nmol/mg protein [41]. The measurement of proteins contained in the sample tissue was performed following the Bradford assay using bovine serum albumin as a reference [42].

### 2.10. Statistical Analysis

All in vitro tests were performed in triplicates, and then the average values were plotted against their standard derivations. Behavioral results were analyzed by one-way ANOVA test. All results are expressed as mean ± SEM of at least four parallel measurements, considering the number of animals in each group (*n* = 4 to 6). *p* values < 0.05 were regarded as significant.

## 3. Results

### 3.1. Total Phenolic and Flavonoid Contents

Total phenolic and flavonoid contents of both extracts of CSP (EtOAc and Acetone extracts) were evaluated. The total phenolics content of CSP extracts was expressed as mg of GAE/g of extract. The acetone extract was more efficient in extracting the almost charge of total polyphenols at about (95.73 ± 0.01 mg of GAE/g of extract), significantly important compared with the ethyl acetate extract, which was barely (30.88 ± 0.07 mg of GAE/g of extract). The rate of total flavonoids was as well consistent in the acetone extract vs. the EtOAc extract, respectively (63.08 ± 0.05 vs. 24.95 ± 0.03 mg of QeE/g of extract).

### 3.2. Antibacterial Activity

The results of MIC and MBC of the antibacterial activity of both extracts EtOAc and Acet extracts are listed in Table 1. It can be seen that both extracts of CSP exhibit significant antibacterial activity against the Gram-positive strains (*Bacillus subtilis* and *Staphylococcus aureus*). Regarding gram-negative strains, the extracts showed an antibacterial effect against *Pseudomonas aeruginosa*, yet *Escherichia coli* was found resistant to both extracts. The MICs of the EtOAc extract ranged from 1.5 to 7.5 mg/mL and MBCs from 3 to 24 mg/mL. Therefore, for the Acet extract, MICs range from 3.125 to 6.25 mg/mL, and MBC from 6.25 to 50 mg/mL. The 2% DMSO was used as a negative control because it does not show inhibition on the targeted strains.

### 3.3. β-Carotene Assay

**Figure 1:**
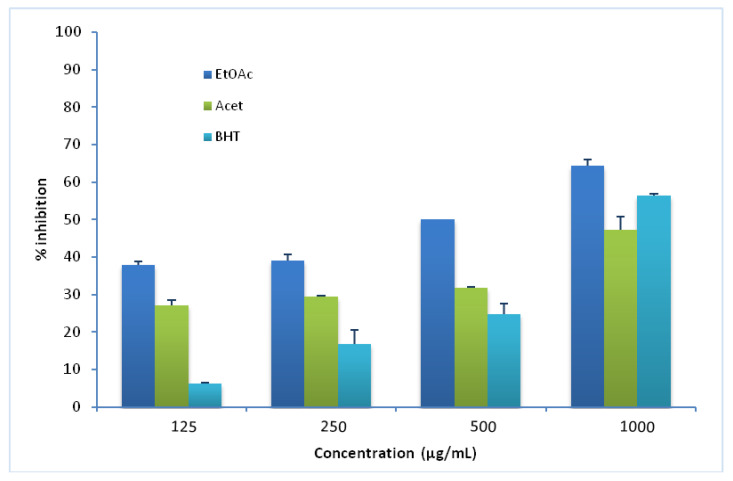
Lipid peroxidation inhibition %/linoleic acid inhibition percentage of the Ethyl acetate (EtOAc) and Acetone (Acet) extracts of Carob seed peels vs. BHT.

β-carotene is an influential antioxidant that has a vital role in protecting against lipid peroxidation in tissues [43]. The inhibitory effect against the loss of coloration of β-carotene is estimated by evaluating the inhibition of the volatile organic compounds and the formation of the conjugated diene hydroperoxides resulting from the oxidation of linoleic acid [44]. As shown in Figure 1, the ethyl acetate extract exhibited a high antioxidant capacity compared with the acetone extract and BHT. Different concentrations were applied. At 1000 µg/ mL, the ethyl acetate extract showed the highest capacity to inhibit the discoloration of β-carotene superior to the BHT (58.3% vs. 46%), followed by the activity of the acetone extract with about (47.25%) inhibition rate. It should be mentioned that a previous study elaborated in our laboratory about the phytochemical screening and antioxidant activity of the same extracts showed that the ethyl acetate extract contained a high level of polyphenols and flavonoids, leading to a strong antioxidant activity stated using the DPPH, FRAP (Ferric reducing power) and PM (phosphomolybdenum assay) tests and in the present study also by using β-carotene test [23].

### 3.4. Y-Maze Test

**Figure 2:**
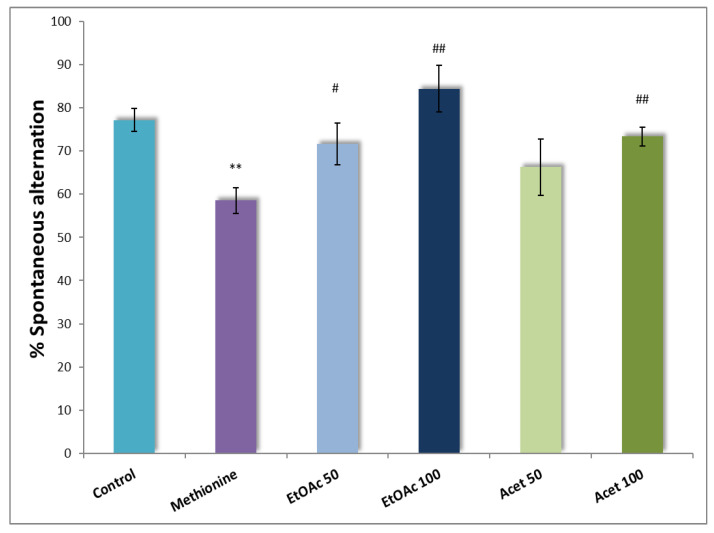
Carob seed peels extract effect) on spontaneous alternation percentage in Y-maze task vs. Control and methionine groups. Data represent the mean ± SEM (*n* = 4–6 per group) with ** *p* ≤ 0.01 vs. Control group, # *p* ≤ 0.05 and ## *p* ≤ 0.01 vs. methionine group.

Oral administration of CSP extracts (50 and 100 mg/kg) for 7 consecutive days to mice displayed a short positive change in spontaneous alternations. As shown in Figure 2, the EtOAc extract increased the correct spontaneous alternation with a significant statistical difference (*p* = 0.001). Thus, at a dose of 100 mg/kg, the ethyl acetate extract increased remarkably the spontaneous alternation (84.37 ± 5.41) compared with the half dose of 50 mg/kg, about (71.62 ± 4.78) vs. methionine group (58.56 ± 2.30). Separately, the same observations were estimated for the acetone extract, with a significant increase (*p* ≤ 0.01) of the spontaneous alternation with 100 mg/kg about (73.34 ± 2.10) compared with 50 mg/kg (*p* ≥ 0.2).

### 3.5. Elevated Plus Maze

**Figure 3:**
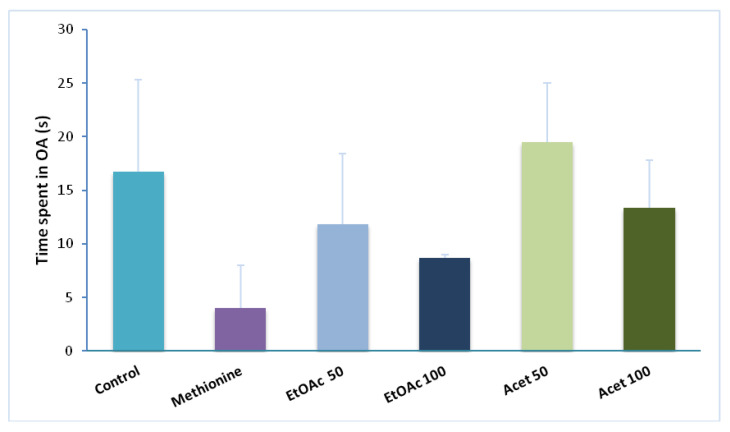
CSP extracts an anxiolytic-like effect represented by the time spent in open arms (OA) by (s) in the elevated plus maze test vs. control and methionine groups. Data represent the mean ± SEM (*n* = 4–6 per group).

The elevated plus-maze is the most commonly applied test for measuring the anxiolytic effect of drugs and also for estimating the spontaneous exploratory behavior of rodents in new areas or environments. As reported in Figure 3, all extracts enhance the time spent in open arms (13.29 ± 2.30 s) compared with the methionine group (4 s). the time spent in open arms decreased clearly in the methionine group (4 s) compared with the control group (19.75 ± 8.56 s). The administration of EtOAc extract (50 mg/kg) exerts an anxiolytic-like effect on the elevated plus maze reflected by a prolonged time passed in open arms (11.83 ± 6.56 s). However, a dose of 100 mg/kg was less effective than the half dose (8.5 ± 0.2 s). The groups administrated with the acetone extract showed independent-dose effects. Thus, both extracts showed the same effects on time spent in the open arms with the same observations in terms of doses administered. Therefore, in this task, the acetone extract was more potent in reducing the level of anxiety than the ethyl acetate extract. What is surprising is the fact that the anova test did not give a significant statistical difference between the groups (*p* > 0.05).

### 3.6. Forced Swimming Test

**Figure 4:**
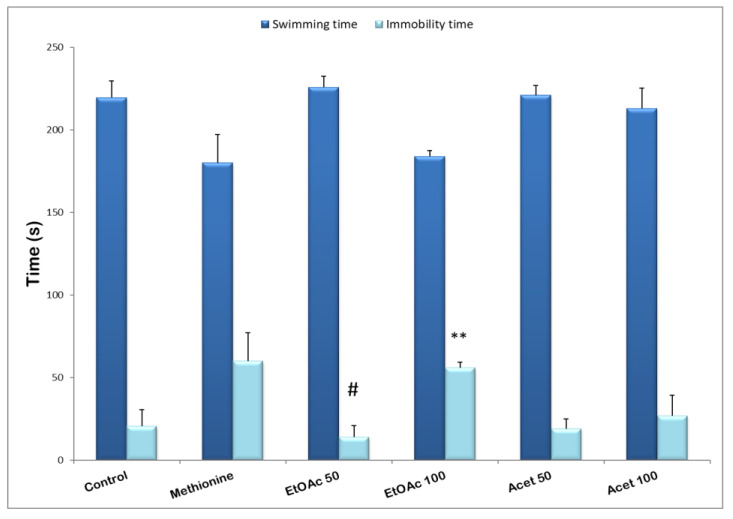
The antidepressant effect of the extracts of carob seed peels extracts (50 and 100 mg/kg) on immobility time and swimming time in mice. Data represent the mean ± SEM (*n* = 4–6 animals) with ** *p* ≤ 0.01 vs. control and # *p* ≤ 0.05 vs. Methionine groups.

In this task, the two used extracts of carob seed peels at various doses decreased the time of immobility compared with the methionine group. The acetone extract significantly shortens the immobility time (14.2 ± 6.78 s, *p* ≤ 0.05) at the minimum dose tested (50 mg/kg), suggesting antidepressant activity compared with the methionine group (60 ± 17.2 s) as well for the ethyl acetate extract (19 ± 5.87, and 27 ± 12.34 s) respectively for 50 and 100 mg/kg. Therefore, with the high dose of the ethyl acetate extract, a small change was detected in the immobility time (56 ± 3.34 s) vs. (60 ± 17.2 s) methionine group, but compared with the control group (20.5 ± 10.21 s) a significant enhance was detected (*p* ≤ 0.01). Nevertheless, the half dose of 50 mg/kg effectively decreased the immobility time, suggesting an interesting antidepressant effect of the EtOAc extract of CPS (see Figure 4).

### 3.7. Novel Object Recognition

**Figure 5:**
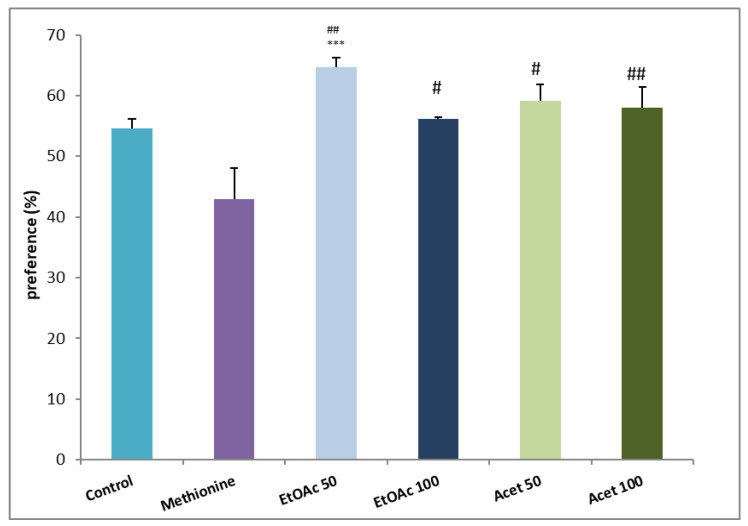
Effects of carob seed peel extracts on the preference percentage of the familiar object vs. the new object compared with control and methionine groups. *** *p* ≤ 0.001 comparing with control group and # *p* ≤ 0.05, ## *p* ≤ 0.01 vs. methionine group.

The administration of both extracts enhanced the exploration time of mice. As observed in Figure 5, the ethyl acetate of both doses compared with the methionine group (64.63 ± 1.55 and 56.11 ± 0.32 vs. 42.89 ± 5.18) enhanced significantly the preference time for the new object (*p* ≤ 0.001 and *p* ≤ 0.05) for 50 and 100 mg/kg respectively. The acetone extract enhances memory retention and the exploratory level of the novel object without an observed difference between the two doses. The most remarkable is that also, compared with the control group, the preference percentage registered a significant increase, especially for those administrated with ethyl acetate extract (54.7% ± 1.66, *p* ≤ 0.001).

### 3.8. Effect of CSP Extracts on SOD Activity

**Figure 6:**
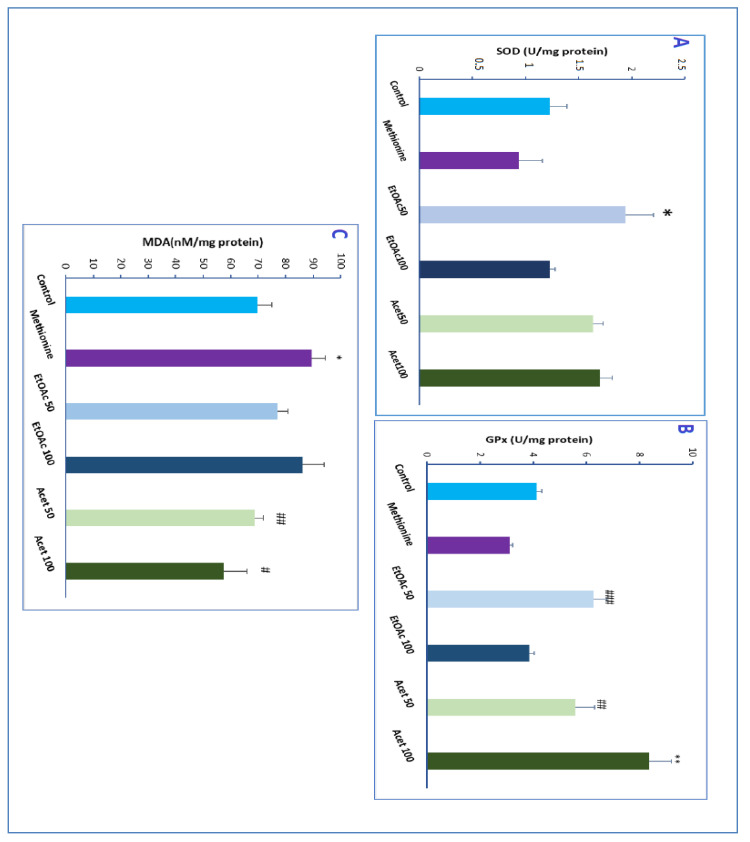
Effect of CSP extracts administration (Ethyl acetate and Acetone (EtOAc 50 and 100 mg/kg) on SOD-specific activity (**A**), GPx-specific activity (**B**), and on MDA level (**C**) vs. methionine and control groups. Values mean ± SEM with * *p* ≤ 0.05, ** *p* ≤ 0.01 compared with control and # *p* ≤ 0.05, ## *p* ≤ 0.01, and ### *p* ≤ 0.001 vs. methionine group.

The SOD activity of different groups of CSP extracts compared with control and methionine groups was reported in Figure 6A. The level of Superoxide increased remarkably and with a significant difference when administrated with ethyl acetate and acetone extracts at a dose of 50 mg/kg, respectively, around *p*-value ≤ 0.02, vs. methionine group. No high difference distinguishable was related to the administration of the two doses of the acetone extract; unexpectedly, the maximum dose of ethyl acetate decreased the SOD activity compared with the 50 mg/kg dose. Therefore, all extracts increased the SOD activity. However, it should be noted that the ethyl acetate extract at a dose of 50 mg/kg showed the highest SOD activity compared with normal mice (*p* = 0.04).

### 3.9. Effect of CSP Extracts on GPx Activity

The CSP extracts significantly enhanced the GPx activity in mice treated with methionine compared with the methionine group (*p* = 0.0015) (see Figure 6B). The ethyl acetate extract was remarkably efficient in increasing the GPx-specific activity at a dose of 50 mg/kg also, a difference in the dose of the acetone extract positively influences the activity, i.e., with increasing the dose, the activity increased. Unlike the acetone extract, the ethyl acetate extract didn’t show an improvement when administrating a high dose (100 mg/kg) compared with the methionine group (*p* ≤ 0.01).

### 3.10. Determination of Malondialdehyde

Regarding the MDA concentration, we detected a significant decrease in groups treated with methionine and administrated with ethyl acetate and acetone extract (*p* ≤ 0.04) compared with methionine extract (see Figure 6C). Groups treated with Acetone extract showed a decreased MDA concentration compared with the control group. Furthermore, a significant decrease was observed with acetone extract at a dose of 100 mg/kg about (*p* ≤ 0.04) compared with the methionine group. In contrast, a decreased concentration of lipid peroxidation was detected at a dose of 50 mg/kg of ethyl acetate extract.

## 4. Discussion

Before interpreting our results, we would just such as to restate our main aims. The first one was to evaluate the effect of CSP extracts, as a natural component exerting an effective antioxidant activity, in the reduction in negative symptoms of schizophrenia induced by methionine. The second one is to give further evidence to support the hypothesis of the correlation of OS damage with the development of neuropsychiatric disorders. The phytochemical analysis of the seed peel showed its richness in secondary metabolites with considerable therapeutic and medicinal effects. Indeed, both extracts revealed high contents of polyphenols and flavonoid compounds. Therefore, the presence of these bioactive compounds in CSP suggests important antioxidant properties of this natural compound, and this was proved by the β-carotene discoloration inhibition test. Our results are in good agreement with previous results reported on the bioactivity of different parts of the carob tree and, in particular, with those related to seed peels [23,45,46].

The MIC and MBC values related to the antibacterial test indicate that both extracts exhibit an inhibitory effect against *Bacillus subtilis*, *Staphylococcus aureus*, and *Pseudomonas aeruginosa* bacterial strains. However, *Escherichia coli* was resistant to both CSP extracts. The ethyl acetate extract was more efficient than the acetone extract against *Bacillus subtilis* with a very low value of MIC (1.5 mg/mL) and MBC (3 mg/mL). Therefore, the Acetone extract performed a remarkable antibacterial activity against *Staphylococcus aureus* at about (3.125 mg/mL vs. 7.5 mg/mL for the Ethyl acetate extract). Previous studies reported the antibacterial activity of carob bean essential oil. Thus, the MIC values against gram-positive strains were about (62 to 1.25 mg/mL), and (1.25 to 2.5 mg mL) for Gram-negative strains [47]. Taking advantage of the resistance of *Staphylococcus aureus* against many phytochemical families [48], the satisfactory result obtained of the antibacterial activity of CSP extracts against gram-positive strains seems to confirm the strong bioactivity of this natural compound.

The current investigation involved oral administration of two dosages of ethyl acetate and acetone extracts of CSP (50 and 100 mg/kg) at the same time for 7 consecutive days. The methionine injection was executed 7 days before extracts administration. After one week, we tested the effect of those extracts on mice with schizophrenic behavior compared to the control group and methionine group (receiving only methionine without any extract).

Behavioral tests are direct practical tools used to analyze and estimate the potential dysfunctions caused by new drug administration. Based on our findings, methionine administration replicated the majority of the negative symptoms of schizophrenia. Thus, we found a decrease in the percentage of spontaneous alternations using the Y-maze test, a decrease in the time spent in the open arms shown in the EPM test, and an increase in the immobility time in the forced swimming test. In addition, using the novel object recognition test, we detected a decrease in the preference percentage of the new object. Thus, the negative symptoms such as anxiety, depression, and memory deficits were recorded, which confirmed the validity of the animal model of schizophrenia used in this study and described in previous research [31,32,49,50].

A Y-maze test is a tool applied to assess the level of anxiety and short-term memory deficits. The data obtained using this test showed that the EtOAc extract increases the spontaneous alternation of mice treated previously with methionine compared with treated mice with sole methionine. Furthermore, in the elevated plus maze task, both CSP extracts significantly enhanced the time spent in open arms. Therefore, we assumed by this test the strong effect of CSP suggesting the anxiolytic effect of both extracts as suggested by previous studies, in which we screened only the anxiety-like effect of the extracts of CSP [23]. Consequently, we assume that the performance wasn’t reduced by the methionine injection. Nevertheless, we confirm the powerful effect of the CSP extracts on short-term memory impairment and anxiety behavior.

The antidepressant effect of CSP on mice with schizophrenic behavior was estimated using the forced swimming test. The experiments carried out during this test are described as a good screening tool with good reliability and predictive validity, which remains one of the most used tools for screening antidepressants [38,44]. Compared to the methionine group, the immobility time was reduced significantly, especially the group administrated with ethyl acetate performed a high antidepressant activity. The carob pod extracts (25 and 50 mg/kg) have previously proven their antidepressant effect in forced swimming tests [51]. Thus, this study confirms that all the parts of the carob tree containing polyphenols ameliorate the immobility time and perform a high antidepressant effect, and accordingly, the correlation between antioxidant activity and amelioration of cognitive impairment is confirmed though. It should, however, be noted that the antidepressant effect of the different extracts depends essentially on the dose used. According to our study, the dose of 100 mg/kg was less effective in reducing the immobility time of mice than the minimal dose of 50 mf/Kg. Therefore, a specific study should be investigated in order to identify the dose that will have the most relevant antidepressant effect.

Novel object recognition enables us to study memory and learning, the preference for novelty, the influence of different brain regions in the process of recognition, and even the study of different drugs and their effects [52]. A significant increase in the preference percentage was observed after administration of both extracts, especially the ethyl acetate extract with a dose of 50 mg/kg. The preference percentage was higher than the methionine group and even that of the control group admitting as well the outstanding effect of CSP on the improvement of short-term, intermediate, and long-term memory.

In this study, we pointed out the negative effect of methionine in enhancing anxiety level and depression, confirming consequently the animal model of schizophrenia induced by methionine. During 7 days of the administration, the methionine injection reduced the spontaneous alternation in the Y-maze test and drastically diminished the time spent in open arms, which is a direct index of anxiety in the elevated plus maze task. Moreover, in the forced swimming test, the methionine group performed a high level of immobility time and reduced swimming activity as well the deficiency in memory detected in the preference percentage in the novel object recognition.

Our findings confirm the suggested hypothesis of the use of natural antioxidants in neuropsychiatric disorders and, in this case, for schizophrenia therapy. Numerous studies have established that polyphenols improve cognitive functions in mice models of neuropsychiatric disorders. For instance, the extracts of *Ginkgo biloba* were found to have a beneficial effect on memory impairment in a range of cognitive disorders [53]. A worthwhile study by Foyet et al. [33] proved the efficient role of the methanol extract of *Hibiscus asper* in managing the neurological abnormalities in Parkinson’s disease conditions. Likewise, the extract of *Emilia coccinae* was found to ameliorate the cognitive dysfunction deficiency induced by scopolamine [54]. All these preliminary results demonstrate compelling evidence of the major contribution of natural antioxidants to remove the oxidative damages firstly and eventually to recover from neurological diseases. Thus, the Measurement of antioxidant enzymes and lipid peroxidation justified this link.

The SOD (a critical enzyme in the detoxification of superoxide radicals and in the reduction in oxidative stress) was reported to be specifically reduced in schizophrenia patients and eventually in untreated patients [11]. Our results are in good agreement with the prior findings. Remarkably the measured SOD activity in the methionine group was found to be reduced compared with the control group. Therefore, the administration of CSP extracts significantly enhanced the SOD activity, also reported to be increased in the schizophrenic patients treated with quetiapine and haloperidol [55,56]. Indeed, the latter was explained by the strong antioxidant activity of these treatments. Hence, this finding confirmed the high antioxidant activity of carob seed peels and the outstanding capacity of this nutritional compound to remedy and regulate the antioxidant system of schizophrenic patients.

The significant decrease in GPx level found in the methionine group in the present study is in accordance with the predicted results and the previously reported studies. The administrated carob seed peel extracts increased the GPx activity, especially the acetone extract. However, using typical or atypical medications, the GPx levels were decreased in the treated schizophrenic patients [57]. Therefore, this was explained with the long-term treatment, which could induce similar effects on the activities of antioxidant enzymes and lipid peroxidation. However, therapy with natural antioxidants will never result in a negative effect on patients with schizophrenia’s view that they are natural and safe.

The levels of the MDA (regarded as the most accurate measures of lipid auto-oxidation level) were increased in the methionine group compared with the control group and as reported for the MDA status of schizophrenic patients. Yet, few researchers have also reported no changes in the MDA level [39]. The MDA level was moderately reduced by the administration of carob seed peels with a small split between the used extracts. Yet, we should notice that even though the levels of MDA for both extracts were reduced compared with the methionine group in the opposite of the usual treatments aforementioned (medications used for schizophrenic patients).

As aforementioned, the CSP exerted a high antioxidant activity related especially to the high content of TF and TF content. Remarkably, these high properties were directly related to a positive effect on cognitive impairment and, consequently, in the negative symptoms of schizophrenia. Our experiments were confirmed as well by the stabilized levels of the antioxidant enzymes; thus, our study provides additional support for the oxidative stress-neuropsychiatric disorders correlation. However, some limitations are worth noting. Some mice have died (2 mice) during the tests due essentially to the problem of acclimatization. Therefore, this doesn’t diminish the quality of the present study. A possible direction was opened, and future work is planned to isolate the pure molecules performing the potential antioxidant, anxiety-like, and antidepressant-like effects and improving the cognitive functions related to dementia or schizophrenia.

## 5. Conclusions

To sum up, our work suggests the protective effect of Carob seed peels against mood disorders and negative symptoms related to the development of schizophrenia. This paper highlighted the importance of antioxidant activity to enhance the cognitive functions of animal models of Schizophrenia induced by methionine. We have obtained satisfactory results proving that the high antioxidant activity and the richness of carob seed peels on phytochemical compounds such as phenols and flavonoids could prevent and remedy the negative symptoms of neuropsychiatric disorders. These findings are promising, and our investigations into this area are still ongoing to validate this direction as effective for protecting and preventing neuropsychiatric disorders using natural compounds from carob seed peels.

## Figures and Tables

**Table 1:** Antibacterial activities of CSP extracts (EtOAc: Ethyl acetate extract, Acet: Acetone extract) expressed as MIC and MBC in mg/mL.

	MIC (mg/mL)		MBC (mg/mL)	
	EtOAc	Acet	EtOAc	Acet
*Bacillus subtilis* ILP1428B	1.5	3.125	3	6.25
*Pseudomonas aeruginosa* ATCC27653	6	6.25	24	50
*Escherichia coli* CIP5412	-	-	-	-
*Staphylococcus aureus* CIP543154	7.5	3.125	7.5	12.5

## Data Availability

All data is available on request.

## Abbreviations

Acet 50: acetone extract 50 mg/kg; Acet 100; acetone extract 100 mg/kg. CSP: carob seed peels; EtOAc 50: ethyl acetate extract 50 mg/kg; EtOAc 100: ethyl acetate extract 100 mg/kg.
